# Supplementary material for: An internal promoter underlies the difference in disease severity between N- and C-terminal truncation mutations of Titin in zebrafish
Source: eLife. 2015 Oct 16;4:e09406. doi: 10.7554/eLife.09406 (PMC4720518; doi:10.7554/eLife.09406)
Supplement: Supplementary file 1. — DOI: http://dx.doi.org/10.7554/eLife.09406.019 [file elife-09406-supp1.zip › TTN_truncations Supplementary File 1 v2.pdf]

Supplementary File 1A

Gene	Disorder
**AARS2**	mitochondrial cardiomyopathy
**ABCC9**	DCM
**ACTA1**	research candidate
**ACTC1**	DCM,HCM,LVNC
**ACTN2**	DCM
**AGK**	mitochondrial cardiomyopathy
**AKAP1**	research candidate
**AKAP1**	research candidate
**AKAP10**	research candidate
**AKAP12**	research candidate
**AKAP13**	research candidate
**AKAP5**	research candidate
**AKAP6**	research candidate
**AKAP7**	research candidate
**AKAP9**	LQT
**ANK2**	LQT
**ANKRD1**	DCM,HCM
**ASPH**	research candidate
**BAG3**	DCM
**CACNA1C**	LQT,Brugada
**CACNA2D1**	Brugada
**CACNA2D2**	research candidate
**CACNA2D3**	research candidate
**CACNB2**	Brugada
**CALM1**	CPVT
**CASQ2**	CPVT
**CAV3**	HCM, LQT
**CMYA5**	research candidate
**COX15**	mitochondrial cardiomyopathy

**CRYAB**	DCM,HCM
**CSRP3**	DCM,HCM
**CTF1**	DCM
**CTNNA3**	ARVC
**DES**	DCM,RCM
**DMD**	DCM
**DNAJC19**	DCM
**DPP6**	VF
**DSC2**	ARVC
**DSG2**	ARVC
**DSP**	DCM,ARVC
**DTNA**	LVNC
**EMD**	DCM
**EYA4**	DCM
**FHL1**	HCM
**FHL2**	DCM
**FKTN**	DCM
**FLNC**	research candidate
**FOXD4**	DCM
**GAA**	Pompe
**GJA1**	research candidate
**GJA5**	SCD
**GLA**	Fabry
**GPD1L**	Brugada
**HCN4**	Brugada
**HRC**	research candidate
**HSPB2**	research candidate
**HSPB6**	research candidate
**HSPB7**	research candidate
**HSPB8**	research candidate
**ILK**	DCM
**JPH2**	HCM

**JUP**	DCM,ARVC
**KCNA5**	research candidate
**KCND3**	research candidate
**KCNE1**	LQT
**KCNE1L**	research candidate
**KCNE2**	LQT
**KCNE3**	Brugada
**KCNE4**	research candidate
**KCNH2**	LQT
**KCNIP2**	research candidate
**KCNJ11**	research candidate
**KCNJ2**	LQT
**KCNJ4**	research candidate
**KCNJ8**	research candidate
**KCNK1**	research candidate
**KCNN2**	research candidate
**KCNN3**	research candidate
**KCNQ1**	LQT
**KLF10**	HCM
**LAMA4**	DCM
**LAMP2**	Danon
**LDB3**	DCM,LVNC
**LMNA**	DCM
**LMOD2**	research candidate
**MIB1**	LVNC
**MRPL3**	mitochondrial cardiomyopathy
**MURC**	HCM
**MYBPC3**	DCM,HCM
**MYH6**	HCM
**MYH7**	DCM,HCM,RCM,LVNC
**MYL2**	HCM
**MYL3**	HCM

**MYLK2**	research candidate
**MYOM1**	research candidate
**MYOT**	research candidate
**MYOZ2**	HCM
**MYPN**	DCM
**NDUFAF1**	mitochondrial cardiomyopathy
**NDUFS2**	mitochondrial cardiomyopathy
**NEBL**	DCM
**NEXN**	DCM,HCM
**NOS1AP**	research candidate
**NPPA**	AF
**OBSCN**	HCM
**PDE3A**	research candidate
**PDE4A**	research candidate
**PDE4B**	research candidate
**PDE4D**	research candidate
**PDE4DIP**	research candidate
**PDLIM3**	DCM
**PKP2**	ARVC
**PLN**	DCM
**PRKAG2**	HCM
**PSEN1**	DCM
**PSEN2**	DCM
**RANGRF**	Brugada
**RBM20**	DCM
**RBM24**	research candidate
**RYR2**	ARVC,CPVT
**SCN10A**	research candidate
**SCN1B**	Brugada
**SCN2B**	research candidate
**SCN3B**	Brugada
**SCN4B**	LQT

**SCN5A**	DCM,Brugada,LQT
**SCN7A**	research candidate
**SCO2**	mitochondrial cardiomyopathy
**SDHA**	DCM
**SGCD**	DCM
**SLC25A3**	mitochondrial cardiomyopathy
**SLC25A4**	mitochondrial cardiomyopathy
**SLMAP**	Brugada
**SNTA1**	LQT
**SVIL**	research candidate
**SYNE1**	DCM
**SYNE2**	DCM
**SYNM**	research candidate
**TAZ**	DCM,LVNC, mitochondrial cardiomyopathy
**TCAP**	DCM,HCM
**TGFB3**	ARVC
**TLN1**	research candidate
**TMEM43**	ARVC
**TMEM70**	mitochondrial cardiomyopathy
**TMOD1**	research candidate
**TMPO**	DCM
**TNNC1**	DCM,HCM
**TNNI3**	DCM,HCM,RCM
**TNNT2**	DCM,HCM,RCM
**TPM1**	DCM,HCM
**TRDN**	CPVT
**TRIM63**	research candidate
**TTN**	DCM,HCM
**TTR**	HCM
**USP13**	research candidate
**UTRN**	research candidate
**VCL**	DCM,HCM

**VIM**	research candidate
**XIRP1**	research candidate
**ZYX**	research candidate

Supplementary File 1B

Primer	Application	Function	Sequence
**Univ_UMI_RACE**	5’RACE	template switching oligonucleotide	CTCAAGCTATGCATCCAACGCGNNNN/ideoxyU/NNNN/ideoxyU/NNNN/ideoxyU/CTTrGrG+G
**dr_e116_rt**	5’RACE	reverse transcription (zebrafish)	TTGGTTCCAGCTTTGTACCC
**mm_e224_rt**	5’RACE	reverse transcription (mouse)	TGGCCTGAGTTTTGAAATCC
**univ_f**	5’RACE	PCR (mouse, zebrafish)	CTCAAGCTATGCATCCAACGCG
**mm_exon224_nest**	5’RACE	PCR (mouse)	AGGGTGAGCGTGTGAACTCT
**dr_exon116_nest**	5’RACE	PCR (zebrafish)	ATTCCACAGGTATGCCCTCA
**hs_mm_exon222/239_f**	Alternative Promoter Analysis	PCR (human, mouse)	TCTAGTGCCCACCTCCGAGTTAA
**mm_altprom_f**	Alternative Promoter Analysis	PCR (mouse)	GAGACAGATGCAGCGCAGATTTCT
**mm_hs_e225/242_r**	Alternative Promoter Analysis	PCR (human, mouse)	GACCCTGCCATCAGCAACAATTTC
**hs_altprom_f_1**	Alternative Promoter Analysis	PCR (human)	GAGACAGATGCAGCACAGATTTCTCG
**dr_e115_f**	Alternative Promoter Analysis	PCR (zebrafish)	GCTAAATCCAAAGCTCAGCTCAGAGTT
**dr_altprom_f**	Alternative Promoter Analysis	PCR (zebrafish)	TCCTGGAACCTGGAGGTGACTAAC
**dr_exon118_r**	Alternative Promoter Analysis	PCR (zebrafish)	AAACCACCTGACCTCTGCTT
**gRNA-n1**	CRISPR/Cas9	gRNA-n1	GGTCAGGAGCAGTGGGAAGA

**gRNA-n2**	CRISPR/ Cas9	gRNA-n2	GGAGACTACAAATT TGTGGC
**gRNA-n3**	CRISPR/ Cas9	gRNA-n3	GGTGATGGTGCCG CCTGTGC
**gRNA-c1**	CRISPR/ Cas9	gRNA-c1	GGCAAACCAGTGA CTTCTGC
**gRNA-c2**	CRISPR/ Cas9	gRNA-c2	GGCCAGCACCTAAA GTTACT
**gRNA-c3**	CRISPR/ Cas9	gRNA-c3	GGTACTGGGGAATT CAGTGA
**gRNA-e113**	CRISPR/ Cas9	gRNA-e113	GGACTACACCGCA GTCGAGA
**gRNA-e114**	CRISPR/ Cas9	gRNA-e114	GGTCCGTCCACTGT ACAGTG
**gRNA-e115**	CRISPR/ Cas9	gRNA-e115	GGCTGTGAAATTAA GGAGGA
**gRNA-e116**	CRISPR/ Cas9	gRNA-e116	GGCTGCGAACTGT CTTATGA
**gRNA-e122**	CRISPR/ Cas9	gRNA-e122	GGAGGCTTTGTAGT CCTCAT
**n1_geno_f**	genotyping (PCR,Cel1), *in situ*	mut-n1	TGCAATAGCCAAAC TCATTGAT
**n1_geno_r**	genotyping (PCR,Cel1), *in situ*	mut-n1	AGGGACAGGCACA TTTTCAG
**n2_geno_f**	genotyping (PCR,Cel1)	mut-n2	GCTCAGAGAGTTCG CATTCA
**n2_geno_r**	genotyping (PCR,Cel1)	mut-n2	CCTTCTCTTCCAGC TGTGCT
**n3_geno_f**	genotyping (PCR,Cel1)	mut-n3	GAATGGGATCACCA CCAACT
**n3_geno_r**	genotyping (PCR,Cel1)	mut-n3	CAAGCCTTCGCTTC CTTACA
**c1_geno_f**	genotyping (PCR,Cel1)	mut-c1	TCATAACTTACCCAA CACTTCCA
**c1_geno_r**	genotyping (PCR,Cel1)	mut-c1	TGAGCCTTCAATAA CCATGACT
**c2_geno_f**	genotyping (PCR,Cel1)	mut-c2	TGAAGATTACCTAA GACTTTGACACG
**c2_geno_r**	genotyping (PCR,Cel1)	mut-c2	CGTGTGTCTGCTCA AAACCA
**c3_geno_f**	genotyping (PCR,Cel1)	mut-c3	CCAGCTCTGATGCA AACAGT

**c3_geno_r**	genotyping (PCR,Cel1)	mut-c3	AGCACCACCATCAG TTCCTC
**n1_as_wt _f**	genotyping (allele-specific)	mut-n1	TAAAGTGTGGCTTC TGTTGCGGTTCTTT GTCATAGAGGTCAC CCT CTTC
**n1_as_mu t_f**	genotyping (allele-specific)	mut-n1	TTTTCTTTGTCATAG AGGTCACCCTGCTC
**n1_as_r**	genotyping (allele-specific)	mut-n1	TAAGGATGTTGCAC CACCAGAGATTG
**n2_as_wt _f**	genotyping (allele-specific)	mut-n2	GTTTCAGCAGATCC AGCCACA
**n2_as_mu t_f**	genotyping (allele-specific)	mut-n2	TTCAGCAGATCCAG CCAA
**n2_as_r**	genotyping (allele-specific)	mut-n2	CCTTCTCTTCCAGC TGTGCT
**n3_as_wt _f**	genotyping (allele-specific)	mut-n3	TTGTCAAATTTCTTA ACTTCGAGTGGTGG TGATGGTGCCGCCA GTG
**n3_as_mu t_f**	genotyping (allele-specific)	mut-n3	TTGTGGTGGTGATG GTGCCGCCTACT
**n3_as_r**	genotyping (allele-specific)	mut-n3	ATGGTTTAGCCCTG GATTTGGAT
**c1_as_wt_ f**	genotyping (allele-specific)	mut-c1	CATCCTTCAGCCAT TCAATCAGTGGCAT TGGCAAACCAGTGA CTTCTG
**c1_as_mu t_f**	genotyping (allele-specific)	mut-c1	TTTGGCATTGGCAA ACCAGTGACTGGC
**c1_as_r**	genotyping (allele-specific)	mut-c1	CGGCTCCAAGTTTG CGTATGCTG
**c2_as_wt_ f**	genotyping (allele-specific)	mut-c2	ACGTGACCCCTCCT TATGACTATGTCAAC ACCAATGCGCTTCC AAGTA
**c2_as_mu t_f**	genotyping (allele-specific)	mut-c2	ATTGTCAACACCAA TGCGCTTCCACCT

**c2_as_r**	genotyping (allele-specific)	mut-c2	GGGTCTTAGTGTCAGAGCAGGATGT
**c3_as_wt_f**	genotyping (allele-specific)	mut-c3	ACTTACCAACTCTTTCAGAACATGCAGTTTCCATACTGCCTTCACTGA
**c3_as_mut_f**	genotyping (allele-specific)	mut-c3	GGTGCAGTTTCCATACTGCCTTCACTAG
**c3_as_r**	genotyping (allele-specific)	mut-c3	TTTAGAGTGGCTGCTGTCAATGCT
**dr_e115/e116_qpcr_f**	qPCR - Cronos to FL ratio	e115/e116	TCAGCTCAGAGTTAAAGCACGA
**dr_e116_qpcr_r**	qPCR - Cronos to FL ratio	e116	CCCTAGGAACCATTCCACAG
**dr_cronos_qpcr_f**	qPCR - Cronos to FL ratio	cronos	CAATGTCTTCTCCTGAATCCAA
**dr_cronos_e117_qpcr_r**	qPCR - Cronos to FL ratio	e117	GTGTGAGATCTGCCTTCAGC
**mm_e222_qpcr_f**	qPCR - Cronos to FL ratio	e222	TTACACAACTGCCGCCTAGA
**mm_e223/e224_qpcr_r**	qPCR - Cronos to FL ratio	e223	CAGACCAATTACCCGTGGTT
**mm_cronos_qpcr_f**	qPCR - Cronos to FL ratio	cronos	AGGCATTTTGTTTCCTCTCG
**mm_e224_qpcr_r**	qPCR - Cronos to FL ratio	e224	CTTCCTTCTGAACGTGTCACC
**n1_outer_f**	NMD estimate	mut-n1	AGGAAGTCTGGTTGAGTTGGCAG
**n1_outer_r**	NMD estimate	mut-n1	GACACCACTATCTCTGGAATAAGCAACT
**n1_nest_f**	NMD estimate	mut-n1	GCATCTTCGCTATGGTCAGGAGC

**n1_nest_r**	NMD estimate	mut-n1	CAGGTCCAAACCGCTTCATCCG
**n2_outer_f**	NMD estimate	mut-n2	TGTTCACTTTGGGGATGTTGGAGACT
**n2_outer_r**	NMD estimate	mut-n2	GATGTGCTTGGTGAACTCAATGACTC
**n3_outer_f**	NMD estimate	mut-n3	GCAAAGCTACCAATGAGGCTGGT
**n3_outer_r**	NMD estimate	mut-n3	TCTTTGGCACCTCAATGAAGAACAAG
**c1_outer_f**	NMD estimate	mut-c1	GCTACAATAAGAGGAAGGCCAGCA
**c1_outer_r**	NMD estimate	mut-c1	TCGGGAATGACGAGGAAGGTC
**c1_nest_f**	NMD estimate	mut-c1	GCGTATGCTGAAACACTTCAAGGG
**c1_nest_r**	NMD estimate	mut-c1	CATCATCCTTCAGCCATTCAATCTTTGG
**c2_outer_f**	NMD estimate	mut-c2	AAGGGTCTTAGTGTCAGAGCAGGA
**c2_outer_r**	NMD estimate	mut-c2	CAGTTGCGTCCAGTCCACCA
**c2_nest_f**	NMD estimate	mut-c2	GCTACAATAAGAGGAAGGCCAGCA
**c2_nest_r**	NMD estimate	mut-c2	TCGGGAATGACGAGGAAGGTC
**c3_outer_f**	NMD estimate	mut-c3	GCTTTAGAGTGGCTGCTGTCAATG
**c3_outer_r**	NMD estimate	mut-c3	ATCAGGTAGCTCAATATCAGGGATTTCAA
**zCronos_probe_F**	in situ	cronos	GAAAGCGCTATGTGGATTTGTAG

**zCronos_probe_T7R**	in situ	cronos	GCGTAATACGACTCACTATAGCTTTGGATTCAGGAGAAGACATTG
**ttna_e4_qpcr_f**	ttna morpholino efficacy	qPCR	TGAGGAAGAAGCCGTACCTG
**ttna_e4_e5_qpcr_r**	ttna morpholino efficacy	qPCR	CGAGCTTCCACTCTCTTCTCC
